# Top stories of 2009

**DOI:** 10.4103/0972-5229.63027

**Published:** 2010

**Authors:** S. K. Todi

**Affiliations:** **From:** Director – Critical Care & Emergency Medicine, AMRI Hospitals, Kolkata, India

## Procalcitonin

### Procalcitonin: A Biomarker for Bacterial Infection

Procalcitonin is being increasingly used as a diagnostic, prognostic tool and in making decisions on the duration of antibiotic therapy. It has been validated in community-acquired infections, but its utility in ICU setting is still uncertain. Two trials addressed this issue last year.[1]

With increasing prevalence of multidrug-resistant bacterial infection in ICU mandating an empirical broad spectrum antibiotic coverage, timely de-escalation and shortening duration of antibiotic therapy is the only way out for decreasing antibiotic pressure. Duration of antibiotic use, at present, is more of an art and depends on clinical response and the physicians' impression of eradication of infection. It is not uncommon for antibiotics to be continued for weeks.

This study was conducted in a surgical ICU. All patients requiring antibiotic therapy based on confirmed or highly suspected bacterial infections, and at least two concomitant systemic inflammatory response syndrome criteria were eligible. Patients were randomly assigned to either a PCT-guided (study group) or a standard (control group) antibiotic regimen. Antibiotic therapy in the procalcitonin (PCT)-guided group was discontinued if clinical signs and symptoms of infection improved and PCT decreased to < 1 ng/ml or the PCT value was >1 ng/ml, but had dropped to 25–35% of the initial value over 3 days. In the control group, antibiotic treatment was applied as a standard regimen over 8 days. In the PCT group (57 patients), the duration of antibiotic therapy was significantly shorter than that compared with those of controls (53 patients) (5.9 ± 1.7 versus 7.9 ± 0.5 days, *P* < 0.001) without negative effects on the clinical outcome. Further studies in a more heterogenous group of patients and analyzing cost effectiveness of this approach need to be studied.[2]

Truly speaking this was not an ICU trial as it was conducted in the emergency department. A total of 1359 patients with mostly severe Lower respiratory tract infection (LRTIs) were selected between October 2006 and March 2008. Patients were randomized to the administration of antibiotics based on a PCT algorithm with predefined cutoff ranges for initiating or stopping antibiotics (PCT group) or according to standard guidelines (control group). The rate of overall adverse outcome was similar in the PCT and control groups. The mean duration of antibiotics exposure in the PCT group was found to be significantly lower. Antibiotic-associated adverse effects were less frequent in the PCT group.

Therefore, at least in patients with severe LRTI presenting to the emergency department, many of which are viral in origin, judicious use of antibiotic guided by procalcitonin level is feasible.[3]

In another study, use of procalcitonin was studied as a diagnostic and prognostic marker in long-staying ICU patients with new onset fever. Median PCT on the day of fever was 1.18 and 0.17 ng/ml for patients with and without proven infections, respectively (*P* < 0.001). The area under the curve for PCT was 0.85 (95% CI: 0.71–0.93), for CRP 0.65 (95% CI: 0.46–0.78), and for WBC 0.68 (95% CI: 0.49–0.81). A PCT level of 1 ng/ml yielded a negative predictive value of 72% for the presence of infection, while a PCT of 1.16 had a specificity of 100%. A twofold increase of PCT between fever onset and the previous day, was associated with proven infection (*P* = 0.001; OR = 8.55; 2.4–31.1), whereas a fourfold increase of PCT of any of the six preceding days was associated with a positive predictive value exceeding 69.65%. A PCT value <0.5 ng/ml on the third day after the advent of fever was associated with a favorable survival (*P* = 0.01).

## Acinetobacter

*Acinetobacter* colonization and infection have become an endemic in ICUs present in India. Even more worrying is the multidrug resistance, including carbapenem resistance nature of this bug. A number of articles which were published last year also highlighted the same phenomenon occurring in eastern European and other developing countries and also as a sporadic outbreak in the west. The common theme among all these studies is the role of infection control policies in containing this outbreak. Multimodal infection control program led by an infection control nurse particularly with emphasis on proper hand hygiene have reduced the colonization rate in some studies. The role of combined treatment with colistin and tigecycline supplemented with nebulized colistin is highlighted in a few studies. Many studies have noted less attributable mortality to this infection, probably signifying that these infections occur in a very sick group of people with a high-mortality rate. Prior colonization with *Acinetobacter* has been identified as the most important predictor of subsequent infection with this organism in one study. Molecular typing has helped in eliminating an outbreak by finding a common source in some studies. Details concerning prevention and control of outbreaks caused by multidrug-resistant strains of *Acinetobacter* are available from the website of UK Health Protection Agency.[4–9]

## Selective Decontamination of Digestive Tract and Selective Oropharyngeal Decontamination

Selective decontamination of digestive (SDD) tract and selective oropharyngeal decontamination (SOD) are not usually practiced in ICUs present in India. There have been mixed reviews on these practices. With increasing Multidrug resistant (MDR) bacterial colonization and infection and exhaustion of new antibiotics, it is time to have a relook into these practices. A number of articles addressed this important practice last year, along with one from Karnad *et al*.[14] of KEM Hospital. They should be applauded for their effort in furthering original research in critical care discipline in India.

An interesting observation from one study was that incidences of Hospital Acquired Infection (HAI) in general wards tended to be higher in patients who had received either SDD or SOD during ICU stay. In an editorial, it was commented that larger studies are needed to demonstrate efficacy of chlorhexidine–SOD to reduce incidence of ventilator-associated pneumonia (VAP). One study explained the differential effect of toothbrushing and 0.12% chlorhexidine or both on incidence of VAP as diagnosed by Clinical Pulmonary Infection Score (CPIS) score on day 3. The results were equivocal with some suggestion that chlorhexidine might be more beneficial than toothbrushing. On the other hand, the study conducted at K.E.M. Hospital, Mumbai, comparing 0.2% chlorhexidine with 0.01% potassium permanganate as the control did not find any difference in VAP rate between these groups. However, they noticed a decrease in incidence of VAP during the study period compared to historical rate, which the authors attributed to good oral care during the study period.

In a major trial published in NEJM on January 1, 2009 5939 patients were enrolled in the study, with 1990 assigned to standard care, 1904 to SOD, and 2045 to SDD. Crude mortality in the groups at day 28 was 27.5%, 26.6%, and 26.9%, respectively. SDD consisted of 4 days of intravenous cefotaxime and topical application of tobramycin, colistin, and amphotericin B in the oropharynx and stomach. SOD consisted of oropharyngeal application only of the same antibiotics. It was concluded that in an ICU population in which the mortality rate associated with standard care was 27.5% at day 28, the rate was reduced by an estimated 3.5 percentage points with SDD and by 2.9 percentage points with SOD.[10–14]

## Autopsy is Not Dead Yet

Postmortem has been the gold standard for final diagnosis in patients dying in hospital. This science has gradually been less utilized due to various reasons. Few studies have looked into postmortem findings of sepsis patients dying in ICU of multisystem organ failure and irreversible shock.

A total of 235 patients were studied between 1997 and 2006 in a surgical ICU. Pathologies were detected in the lungs (89.8%), kidneys/urinary tract (60%), gastrointestinal tract (54%), cardiovascular system (53.6%), liver (47.7%), spleen (33.2%), central nervous system (18.7%), and pancreas (8.5%). In 180 patients (76.6%), the autopsy revealed a continuous septic focus. The most common continuous foci were pneumonia (41.3%), tracheobronchitis (28.9%), peritonitis (23.4%), uterine/ovarial necrosis (9.8% of female patients), intra-abdominal abscesses (9.1%), and pyelonephritis (6%). A continuous septic focus was observed in 63 of the 71 patients (88.7%), who were admitted to the ICU because of sepsis/septic shock and treated for more than 7 days. The message from this unique study is that even with the present day technology, a continuous focus of infection remains the main cause of patient demise.[15]

## Bugs are Everywhere

Intensive care units have become the hub of multidrug-resistant bacteria. Cross transmission of these bugs is the most common cause of nosocomial infection in the ICU. Even with the best efforts in infection control, it is becoming impossible to eradicate bacteria from the ICU environment. Two studies addressed this issue. Out of the 24 ICU bedside stethoscopes tested, two diaphragms and five earpieces were colonized with pathogenic bacteria. Methicillin resistant *Staphylococcus aureus* (MRSA) cultured from one earpiece persisted after cleaning. Three out of the 22 personal stethoscope diaphragms and five earpieces were colonized with pathogens. After cleaning, two diaphragms and two earpieces were still colonized, demonstrating the importance of regular cleaning. Mobile phones have been found to be contaminated with pathogenic bugs in 9–25% of cases.[16,17]

## Quality Control in ICU

With increasing incidence of nosocomial infections and device-related infections, the only way to decrease this menace would be to have a firm grip on quality control processes in the ICU. Central line-related infections cause a high mortality. Till now emphasis was given only on quality control measures to be taken during insertion of such catheters to decrease subsequent infection. In a recently published study, educational measures and hand hygiene measures have been discussed. The incidence of catheter related blood stream infection (CRBSI) decreased from 3.9 per 1000 catheter days in the preintervention phase to 1.0 per 1000 catheter days in the intervention phase (*P* < 0.001). The other highlight of the study was that education not only increased the compliance with hand hygiene, but also significantly improved the rate of correct performance of the practice. Surviving sepsis campaign has increased the awareness of sepsis management among healthcare providers. Studies are coming out emphasizing its impact, when correctly practiced, on the outcome of this highly fatal syndrome.

In a study carried out in Italy, compliance to five resuscitation and four sepsis management interventions and in-hospital mortality were measured following an educational program on sepsis for physicians and nurses of all hospital departments, and hospital implementation of a specific protocol for the recognition and management of patients with severe sepsis/septic shock, including an early consultation by a skilled “sepsis team.” During the study period, the compliance to all nine interventions increased from 8% to 35% of the patients (*P* < 0.01). The implementation of resuscitation and management interventions were associated with a lower risk of in-hospital mortality (23% vs. 68% and 27% vs. 68%, *P* < 0.01). In the latter two semesters, after activation of the “sepsis team,” in-hospital mortality of ICU septic-shock patients decreased by about 40% compared with those of the previous period (32% vs. 79%, *P* < 0.01). This kind of impressive result has also been replicated in other centers. This study highlights that maximum efforts should be made to implement such programs in our hospitals to bring research to the bedside for the benefit of our patients.

Having a mandatory checklist of things to be done, definitely increases efficiency in ICU practice. This practice is essential in safe industries like the aviation and automobile industry. Verbal handover is still the prevalent practice in healthcare. Daily checklist pioneered by Dr. Pronovost from John Hopkins has revolutionized the quality control program in the ICU. A study conducted in a surgical ICU looked at this practice. Bedside consideration improved on the use of deep venous thrombosis prophylaxis (*P* < .05), stress ulcer prophylaxis (*P* < 0.01), oral care for ventilated patients (*P* < 0.01), electrolyte repletion (*P* < 0.01), initiation of physical therapy (*P* < 0.05), and documentation of restraint orders (*P* < 0.0001).[18–20]

## Epidemiology

A major study published in 2009 ‘The Extended Prevalence of Infection in Intensive Care (EPIC II)’, a 1-day, prospective, point prevalence study with follow-up was conducted on May 8, 2007. Demographic, physiological, bacteriological, therapeutic, and outcome data were collected for 14,414 patients in 1265 participating ICUs from 75 countries on the study day. On the day of the study, 7087 of 13,796 patients (51%) were identified as infected; 9084 (71%) were receiving antibiotics. The infection was of respiratory origin in 4503 patients (64%), and microbiological culture results were positive in 4947 (70%) of the infected patients; 62% of the positive isolates were gram-negative organisms, 47% were gram-positive, and 19% were fungi. Patients who had longer ICU stays prior to the study day had higher rates of infection, especially infections due to resistant *Staphylococci, Acinetobacter, Pseudomonas* species, and *Candida* species. The ICU mortality rate of infected patients was more than twice that of noninfected patients.[21]

## Can Nosocomial Infection Risk Be Predicted?

The present paradigm of sepsis being conceptualized as an overwhelming immune response does not explain the high incidence of secondary infection in this population which is a marker of immunosuppression. It looks like the phase of immunostimulation is transient and the critically ill patients go into a phase of immunoparalysis which makes them prone to nosocomial sepsis. The challenge has been how to identify these patients with immunosupression who do not fulfill the classical indicators such as neutropenia. Monocytic HLA-DR expression on circulating monocytes (mHLA-DR) in intensive care patients is being studied as a marker of immunosuppression. Early mHLA-DR was decreased in the whole population, however, more deeply in sepsis, the lower the slope of mHLA-DR recovery, the higher the incidence of the first secondary infection. In future therapies, targeting to improve HLA-DR expression might help in preventing nosocomial infection to some extent.[22]

## Pharmacokinetic/Dynamic

There has been recent trend of using beta-lactam antibiotic as a continuous infusion based on a sound physiological principle. Robust outcome studies based on this principle is lacking. A review of all studies looking into trials of continuous infusion vs. standard bolus dose was conducted last year. Among a total of 59 potentially relevant studies, 14 Randomised controlled trial (RCTs) involving a total of 846 patients from nine countries were deemed appropriate for meta-analysis. The use of continuous infusion of a beta-lactam antibiotic was not associated with an improvement in clinical cure or mortality. All RCTs except one used a higher antibiotic dose in the bolus administration group. Two observational studies, not pooled because they did not meet *a priori* criteria for meta-analysis, showed that beta-lactam administration by extended or continuous infusion was associated with an improvement in clinical cure. The difference in the results between the meta-analysis results and the observational studies could be explained by the bias created by a higher dose of antibiotic in the bolus group in the RCTs, and because many of the RCTs only recruited patients with a low acuity of illness. It looks like that jury is still out on the practice of continuous infusion of antibiotics.

Colistin and polymyxin B are being increasingly used in ICUs for multidrug-resistant gram-negative sepsis. The *pk*/*Pd* of these drugs is not studied extensively. In an elegant study, blood levels of colistin were measured after a bolus dose. From this study it looks like we are probably underdosing colistin at the present time, and an initial high-dose bolus might be useful to reach a steady-state earlier.[23,24]

## Unusual Bugs in ICU

Cytomegalovirus is the most common opportunistic viral infection in immunocompromised patients. However, recent studies have demonstrated active cytomegalovirus infection in nonimmunosuppressed ICU patients. In a recent review, active cytomegalovirus infection was found to occur frequently in nonimmunosuppressed patients who stay in the ICU, especially in those with positive cytomegalovirus serology, ICU stay ≥ 5 days, severe sepsis, and high disease severity, in whom the rate of cytomegalovirus infection was up to 36%. Mortality rate has significantly doubled with cytomegalovirus, but a cause-effect relationship has not been established as yet. A large prospective cohort study is needed to define who is at the highest risk for developing active cytomegalovirus infection and to determine its effects on mortality.[25]

## Device-Associated Infection

Ventilator-associated pneumonia is the leading cause of nosocomial sepsis and mortality in ICUs worldwide. Multiple studies addressed various aspects of this infection.

In an elegant study, Falagas *et al*.[27] from Greece looked into the use of colistin nebulizer in VAP due to MDR gram-negative bacteria. The authors used concurrent systemic therapy with antibiotics reported to be resistant to the organism. As colistin disrupts cell wall, it is possible that nebulized colistin, a safer way of delivering this toxic drug acts synergistically with systemic antibiotics. The study considered few patients only, and further research is needed.

Impact of the administration of probiotics on the incidence of ventilator-associated pneumonia: A meta-analysis of randomized controlled trials was studied: Administration of probiotics was associated with lower incidence of ventilator-associated pneumonia than control.

Transfusion and pneumonia in the trauma intensive care unit: an examination of the temporal relations reflected transfusions received after pneumonia rather than etiologically relevant transfusions received before the onset of pneumonia. Transfusion of exclusively older blood, however, increased the risk of pneumonia, further suggesting the importance of blood age with respect to outcomes in trauma patients.[26–31]
